# Using dynamic cell communication improves treatment strategies of breast cancer

**DOI:** 10.1186/s12935-021-01979-9

**Published:** 2021-05-25

**Authors:** Zhibo Liu, Song Hu, Zehui Yun, Wanshan Hu, Shuhua Zhang, Daya Luo

**Affiliations:** 1 Second Clinic Medical College, The Second Affiliated Hospital of Chongqing Medical University, 76 Linjiang Road, Yuzhong District, Chongqing, People’s Republic of China; 2 Thrombosis Center, National Center for Cardiovascular Diseases, Fuwai Hospital, Chinese Academy of Medical Sciences and Peking Union Medical College, Beijing, 100037 China; 3 Queen Mary School, School of Medicine, Nanchang University, Nanchang, People’s Republic of China; 4 School of Medicine, Forth Clinic Medical College, Nanchang University, Nanchang, People’s Republic of China; 5 Jiangxi Cardiovascular Research Institute, Jiangxi Provincial People’s Hospital Affiliated to Nanchang University, Aiguo Road, No. 152, Nanchang, 330006 Jiangxi People’s Republic of China; 6 Department of Biochemistry and Molecular Biology, School of Basic Medical Sciences, Nanchang University, Bayi Road, No. 461, Nanchang, 330006 People’s Republic of China

**Keywords:** Breast cancer microenvironment, Cell communication, Dynamic change, External environment, Therapeutic efficacy

## Abstract

Several insights from the clinical treatment of breast cancer patients have revealed that only a portion of patients achieve the expected curative effect after traditional targeted therapy, that surgical treatment may promote the development of cancer metastasis, and that the optimal combination of neoadjuvant chemotherapy and traditional treatment is not clear. Therefore, a more precise classification of breast cancer and selection of treatment methods should be undertaken to improve the efficacy of clinical treatment. In the clinical treatment of breast cancer, cell communication molecules are often selected as therapeutic targets. However, various cell communications are not static. Their dynamic changes are related to communicating cells, communicating molecules, and various intertwined internal and external environmental factors. Understanding the dynamic microenvironment can help us improve therapeutic efficacy and provide new ways to more accurately determine the cancer status. Therefore, this review describes multiple types of cellular communication in the breast cancer microenvironment and incorporates internal and external environmental factors as variable signaling factors in cell communication. Using dynamic and developmental concepts, we summarize the functional changes in signaling molecules and cells to aid in the diagnosis and treatment of breast cancer.

## Background

The breast cancer microenvironment consists of mammary ductal epithelium, many kinds of stromal cells (such as fibroblasts, immune cells, adipocytes and endothelial cells) and the extracellular matrix (ECM). Stromal cells surround the cancerous ductal epithelial cells and contact each other through the ECM [1, 2]. During breast cancer development, frequent communication exists not only in various kinds of cells in the microenvironment but also between the primary microenvironment and metastatic niche, local microenvironment and internal and external environments of the body.

Cell communication refers to the process in multi-cellular organisms in which cells send a signal to target cells that interact with receptors to cause specific biological effects in target cells. Through accurate and efficient cell communication, physiological and biochemical reactions in cells are regulated and unified to adapt to the changing external environment [3]. Notably, some kinds of cell communications and molecules play dual roles during breast cancer development. Understanding the dynamics of cell communication in the breast cancer microenvironment is helpful for solving certain problems, such as "why histologically identical cell types or the same signaling molecules have different or even opposite effects in tumor progression " and "why the same drug in different stages of tumor progression has different or even opposite functions", and give us a chance to consider potential functions of external environmental factors in the clinical individualized diagnosis and treatment of tumors. Moreover, realizing the real time detection of changes in cell communication will aid in breast cancer treatment.

## Cell communication in the breast cancer microenvironment

According to whether there is physical contact between communicating cells, cell communication can be divided into direct communication and indirect communication. In the breast cancer microenvironment, these two cell communication modes can occur in the same type of cell or between different types of cells.

### Direct cell communication in the breast cancer microenvironment

Direct cell communication in the breast cancer microenvironment mainly consists of gap junctions and contact signaling by plasma-membrane-bound molecules [4]. They exist in the same or heterogeneous type of cells depending on the signaling molecules located on the surface of the cell membrane. The components of gap junctions are not only key cell junction molecules but are also involved in the exchange of information between cells. Contact signaling by plasma-membrane-bound molecules is mediated by cell adhesion molecules, such as proteoglycan or glycoproteins, across the cell membrane. Histologically adjacent and non-adjacent cells that may contact each other under physiological or pathological conditions can specifically recognize and interact with each other to send signals through membrane surface adhesion molecules. This type of communication includes adhesion molecules that participate in cell junctional communication, such as the adhesion belt and desmosomes, and non-cell junctional communication.

#### Gap junction intercellular communication (GJIC)

Gap junctions, which are composed of connexons, consist of connections between histologically identical or heterogeneously adjacent cells. Gap junctions form channels through the pairing of two connexons located in neighboring cells. Human breast ductal epithelial cells, myoepithelial cells and endothelial cells express multiple subtypes of connexin proteins, among which the most important are Cx26 and Cx43. A previous study showed that the deregulated expression of Cx43 and Cx26 in breast cancer often affects the stability of intercellular gap junctions and deregulates cell proliferation. The import of exogenous Cx26 into MCF-7 breast cancer cells can promote the formation of gap junctions in the microenvironment to inhibit the proliferation and invasiveness of breast cancer cells [5]. Macrophages have a variety of subtypes, including nonactivated, classically activated (M1) and alternatively activated (M2) types. Researchers used CMTMR to label M2 macrophages, and CFDA to label MDA-MB-231 cells. They found that the exchange of substances and cell communications were carried out between these cells by GJIC resulting in reduced proliferation and cycling quiescence [6]. Cx has long been regarded as a tumor inhibitor [7]. However, it is more likely that during different periods of tumor development, the expression of Cxs requires dynamic changes to adapt to the progression of cancer. For example, the expression of Cx43 differs during different developmental periods of breast cancer. Cx43 is down-regulated in the primary tumor and induces the loss of GJIC. As cancer progresses, tumor cells regain Cxs expression and maintain GJIC with endothelial barriers to induce extravasation and adherence to the metastatic site. Downregulation of intercellular adhesion helps cancer cells escape from the original site, and then cell aggregation is required to avoid anoikis. Studies have shown that in the early aggregation stage of MCF-7 cells, the rapid establishment of functional gap connections further promotes their aggregation. This observation was confirmed by two gap connection inhibitors (Tonabersat and Meclofenamate) and suggests that connexons may accurately reflect the stages of cancer development [8]. Moreover, members of the Cx family can release ATP and glutamate to independently regulate cell proliferation in GJIC [9]. A recently published study also indicated that Cx26 can form a signaling complex with the pluripotency transcription factor NANOG and focal adhesion kinase (FAK) and activate its downstream signals to maintain stem cell characteristics in cancer cells [10]. These findings suggest more diverse and complex roles of Cxs and gap junctions in breast cancer.

#### Adhesion molecule-mediated cell junction communication: adhesion belts and desmosomes

In adjacent cells, with the help of Ca^2+^, the same types of cadherins can interact with each other to form adhesive belts and desmosomes [11]. These structures not only play a role in resisting mechanical stress and stabilizing tissue integrity but are also involved in cell differentiation, development and migration by mediating cell communications. The adhesion belt includes many intracellular anchoring proteins (catenin, vinculin and α-actinin) that link cadherin to intracellular actin. The main components of desmosome are the armadillo protein and plakin protein families, which link cadherin to intracellular intermediate filaments [12]. E-cadherin plays an important role in the development of breast cancer by recruiting β-catenin to the cytoplasm and forming a complex with it [13]. Studies have shown that E-cadherin inhibits breast cancer cell proliferation, epithelial-mesenchymal transition (EMT) and metastasis by combining with β-catenin and reducing β-catenin transcriptional activation of the target gene cyclin D1 in the nucleus [14-16]. Concomitantly, through the endocytosis of vesicles containing E-cadherin and further through lysosomal digestion, the adhesion ability of breast cancer cells is inhibited, and their migration ability is promoted [17]. Therefore, E-cadherin is often used as an epithelial phenotype marker to detect tumor EMT and metastasis. The changing expression of anchoring proteins in cells is also closely associated with the invasive ability of breast cancer. Vinculin and α-actinin are important components of the adhesive belt. Sejal Desai’s group found that vinculin and α-actinin are up-regulated in radio-resistant MCF-7 cells. The latter can compete with E-cadherin to combine with β-catenin and stimulate downstream AKT/GSK3β signaling pathways. This pathway can then induce the EMT of breast cancer cells and improve their invasive and metastatic abilities [18]. Another study showed that down-regulation of the expression of plakoglobin in breast cancer MCF-7 and T47D cells could reduce intercellular adhesion and promote the migration of breast cancer cells and transfer into blood vessels [19]. Some studies have found that the oncoprotein ErbB2 can down-regulate Perp which is a component of desmosomes, to avoid the anoikis of breast epithelial cells, and in normal breast epithelial cells, detachment-induced Perp upregulation can promote the anoikis of breast epithelial cells [20].

#### Adhesion molecule-mediated non-cell junction communication

In addition to adjacent cells, cell communication also occurs between cells that are not adjacent through direct cell-cell contact; for example, CD24 expressed by tumour cell can interact with the inhibitory receptor sialic-acid-binding Ig-like lectin 10 (Siglec-10), which is expressed by tumour-associated macrophages to send anti-phagocytic signal [21]. This kind of cell communication is dependent on adhesion molecules located on the cell membrane surface. In this way, adhesion molecules, such as selectins and those of the integrin family, mediate cell communications between leukocytes and endothelial cells. The immunoglobulin superfamily (IGSF) plays important roles in cell communication between lymphocytes, antigen presenting cells (e.g., macrophages, dendritic cells) and other cells such as tumor cells. During the development of breast cancer, communication among these cells may be altered. For example, sialyl-Lewis on the surface of breast cancer cells can directly interact with E-selectins on the endothelial cell surface, promoting cancer cell adhesion to endothelial cells and contributing to metastasis to other organs. In the bone, E-selectins are important structural components, and breast cancer cells with elevated expression of sialyl-Lewis can metastasize to the bone by specifically recognizing and combining with bone E-selectins [22]. Direct communication between T cell receptors (TCRs) and major histocompatibility complex (MHC) molecules mediate the activation of T cell immune responses by antigen presentation. Intercellular cell adhesion molecule-1 (ICAM-1) is a member of the IGSF that can be used by regulatory T cells to directly contact Vα24^+^ NKT cells and suppress their immune-killing effects to facilitate immune escape in breast cancer [23]. MHC-I molecules on the surface of immature myeloid cells can directly interact with TCRs located on the surface of T lymphocytes and neutralize the toxicity of T lymphocytes. The use of a TCR mimic to competitively interact with MHC-I located on the surface of breast cancer cells can inhibit their proliferation [24].

### Indirect cell communication in the breast cancer microenvironment

Indirect cell communication includes soluble chemical signal cell communication and extracellular matrix mediated cell communication. The former can mediate paracrine and autocrine communications, and transmit communication molecules between cells through extracellular vesicles, while the latter mainly forms interaction links through the extracellular matrix between cells.

#### Cell communications depending on paracrine and autocrine mechanisms

During indirect cell communication, chemical signal communication mediated by autocrine and paracrine mechanisms has been more commonly and thoroughly studied than that mediated by other mechanisms. Traditionally, these chemical molecules include interleukin (IL), interferon (IFN), tumor necrosis factor (TNF), transforming growth factor (TGF), chemokines and growth factors. It has been confirmed that all cells in the breast cancer microenvironment can release various cytokines and act specifically on surrounding cells by binding to the receptors expressed on their membrane surface. Subsequently, they sequentially induce biological behavioral changes in recipient cells and regulate the occurrence and development of breast cancer through the kinase signaling system. The most abundant cell type in the breast cancer microenvironment is the fibroblast. Fibroblasts can become cancer-associated fibroblasts (CAFs) after communicating with cancer cells [25]. In the breast cancer matrix, nearly 80% of fibroblasts secrete a variety of growth factors, cytokines, proteases and hormones that stimulate tumor cells to obtain an invasive phenotype by the paracrine mechanism to promote the growth, invasion and metastasis of breast cancer. Different types of cells are recruited to the local breast cancer microenvironment through this way of cell communication. A study found that 50 to 96% of women with breast cancer gained weight during treatment. Obese adipose tissue increases TNF-α and IL-6 to promote insulin resistance, insulin and IGF-1 to facilitate cancer cell proliferation. That also increases the risk of breast cancer occurrence by reducing the ratio of adiponectin to leptin, and the release of free fatty acids continuously activates NF-kB pathway, which causes a chronic inflammatory environment and promotes the development of cancer [26]. Tumor cells, dendritic cells, and macrophages in the breast cancer environment secrete chemokines such as CCL5, CCL17, CCL22, and CXCL12, which attract peripheral regulatory T cells (Tregs). Tregs secrete TGF-β which induces the secretion of vascular endothelial growth factor (VEGF) from endothelial cells to facilitate angiogenesis. In a mouse orthotopic implantation model, researchers found that mesenchymal stem cells (MSCs) were recruited to primary breast tumors and facilitated breast cancer cell migration to lymph nodes and lungs via a hypoxia-inducible factor-dependent mechanism. During this process, tumor cells and MSCs express placental growth factor (PGF) and its cognate receptor VEGFR1. This expression pattern has a dual effect by not only promoting the metastasis of breast cancer cells but also inducing the homing effect of MSCs to tumor tissues [27]. It is worth noting that this kind of cell communication can even promote the transition of breast cancer cells to the aggressive phenotype. In breast cancer, Epsin 3 (EPN3) can enhance E-cadherin endocytosis, activate β-catenin/TCF4-mediated EMT and promote the autocrine loop mediated by TGFβ, and ultimately promote metastasis of breast cancer cell. In addition, EPN3 is detected at the invasive front of cancer cells and can independently predict the recurrence of metastatic cancer [28].

#### Cell communication via extracellular vesicles (EVs)

EVs refer to bilayer membrane vesicular bodies that detach from the cell membrane or are secreted from cell. They comprise mainly microvesicles (MVs) and exosomes (Exs). MVs are small vesicles that detach from the membrane after cell activation, damage or apoptosis. They have a diameter of approximately 100–1000 nm. Exs are formed when multi-vesicular bodies fuse with the plasma membrane and can also be released directly from the plasma membrane. They have a diameter of approximately 40–100 nm [29]. Currently, EVs are believed to be released from a variety of cells and to transfer proteins, lipids, nucleic acids and metabolites from donor to recipient cells, thus playing a vital role in cell communication. Breast cancer cells secrete MVs, and Hsp90 on their membrane surface can bind to an ~ 90 kDa crosslinked form of VEGF (VEGF90K) to continuously stimulate VEGFRs in endothelial cells and promote angiogenesis. In this case, signal transduction is insensitive to VEGF antibodies such as Bevacizumab. However, using Hsp90 inhibitors, re-establishes the sensitivity of this signal transduction and inhibit angiogenesis, suggesting that MVs are involved in the development of drug resistance in tumors. [30] In vitro studies have shown that the Exs from MDCK cells contain amphiregulin, which is more stable than other ligands of EGFR and can more effectively promote the invasiveness of breast cancer cells [31]. Jang JY et al reported that the treatment of 4T1 breast cancer cells with epigallocatechin gallate increased the expression of miR-16 and that miR-16 could be secreted via Exs transfer to tumor-associated macrophages, reducing their infiltration and suppressing their polarization toward the M2 phenotype. In this way, miR-16 can inhibit tumor growth [32]. Stromal cells in the tumor microenvironment can also secrete Exs to participate in cell communication. Studies have shown that dendritic cells secrete Exs containing MHC I and II and CD86. Such Exs can be applied to T cells and promote proliferation and enhance anti-tumor immunity [33, 34]. Sarah A Bliss’ team found that dormant breast cancer cells could induce MSCs to release miR-222/223 engulfed by Exs to promote breast cancer cell entry into the dormant state and improve drug resistance. Thus, they created a miR-222/223 antagonist to target dormant breast cancer cells, to improve breast cancer cell sensitivity to carboplatin-based therapy [35]. Some studies have suggested that exosomes in oral saliva are less interfered by other substances than those in the serum, and their acquisition via oral saliva is more convenient and non-invasive. Its value as a cancer biomarker has been found in oral cancer and pancreatic ductal carcinoma, suggesting the potential of exosomes as biomarkers in breast cancer [36].

#### Extracellular matrix-mediated cell communication

The ECM, which plays an important role in mediating cell communication, is composed of macromolecules that are synthesized and secreted by cells and located on the surface of or between cells [37, 38]. The ECM can release or return a large number of cytokines and coordinate with them to regulate different cell biological behaviors. For example, as the most abundant cells in the microenvironment, fibroblasts can use cytomembrane syndecan-1 to cooperate with matrix molecules, such as heparan sulfate (HS) and fibroblast growth factors (FGFs), to combine with cognate receptors, such as FGFR, on the surface of breast cancer cells and assemble into signaling complexes to promote breast cancer cell proliferation via Wnt signaling pathways [39, 40]. On the other hand, a portion of the extracellular matrix can bind specifically to trans-membrane adhesion molecules, such as cadherin, integrin, CD44 or CD36, as a "bridge" to transfer signals between different cells. Thrombospondin (THBS) is expressed by a variety of cells, such as fibroblasts, macrophages and cancer cells [41, 42]. THBS1 inhibits angiogenesis by binding to CD36 on endothelial cells and inducing apoptosis. It is interesting that THBS1 has been shown to inhibit primary tumor growth via antiangiogenic mechanisms but promotes lung metastasis in mouse models, in which it induces the migration and invasion of breast cancer cells via the activation of TGF-β and up-regulation of the urokinase plasminogen activator (uPA) system [43]. Based on studies of the ECM, the breast cancer stroma is typically stiffer than the normal stroma [44]. Mechanical compression can increase the migratory behavior of tumors through the enhanced expression of fibronectin [45]. In MCF10DCIS cells and Eph4Ras cells, researchers found that as breast cancer progresses, the increasing stiffness of ECM, which further induces EMT, invasion and metastasis of cancer cells through the EPHA2/LYN/TWIST1 mechanotransduction pathway [46]. Cancer stem cells (CSCs) have the ability to self-renew, and grow slowly during the dormant state. This makes them insensitive to ordinary chemotherapy and plays an important role in the initiation, metastasis, drug resistance and recurrence of cancer. When CSCs recover from the dormant state, they usually acquire genetic traits and drug resistance [47]. Some researchers have also reported that an increased ECM stiffness can lead to an increase in cancer stem cell (CSC) numbers and higher mobility compared with cells cultured in a softer ECM [48]. Some ECM remodeling enzymes, such as matrix metalloproteinases (MMPs), heparanase and others, can break down the basement membrane surrounding the mammary gland epithelium, which provides room and migration paths for cancer cells and frees ECM-bound growth factors to promote breast cancer metastasis [49].

## Cell communication between the local breast cancer microenvironment and the internal and external environments

In brief, breast cancer is the integrated result of a variety of adaptive responses produced by the body after communicating with internal and external environmental factors. Internal and external environmental factors affect the local tumor microenvironment through various modes of cell communication, and during the process of tumor metastasis, close cell communication also forms between the microenvironment of the primary tumor and the metastatic microenvironment. Therefore, basic research on breast cancer should not only focus on cell communication within the local primary breast cancer microenvironment but also be extended to cell communication between the primary cancer microenvironment and the internal and external environments.

### Cell communications between primary tumors and metastases

With continuous growth of the tumor, tumor cells may be transferred to distant sites to form metastases via the blood, lymph or nerve fibers. The formation and development of secondary tumors is often a major cause of cancer-related death. The lung, bone, liver, and brain are common sites of breast cancer metastasis. This kind of organ selectivity is not only associated with the blood supply but also with cell communication between primary tumors and metastases. For example, researchers have summarized 5 fundamental steps during breast cancer metastasis to the bone: (a) Primary cancer cells secrete soluble factors or microvesicles that cause preliminary metastatic ECM deposition to occur; (b) bone marrow-derived cells (BMDCs) infiltrate the metastatic microenvironment; (c) cancer cells arrive at metastases; (d) micro-metastases develop; (e) metastases form [50].

The metastatic microenvironment, also called the metastatic niche, often depends on the formation of direct communication and indirect cell communications between the primary tumor and metastases. During pulmonary metastasis of breast cancer, the cancer cells can secrete TGF-β3 to promote periostin (POSTN) produced in SMA^+^/VIM^+^ lung fibroblasts to facilitate the formation of metastases [51]. During bone metastasis of breast cancer, E-cadherin expressed on luminal breast cancer cells and N-cadherin expressed on osteoblasts can interact with cell surface molecules to enhance breast cancer cell proliferation. In addition, bone metastases of breast cancer cells can secrete certain cell factors to directly stimulate osteoclastogenesis or indirectly stimulate osteoblast-secreted cytokines, osteoclastogenic factors to enhance osteoclastic activity to adapt to the formation of the metastatic microenvironment [52]. During brain metastasis of breast cancer, cancer cells can secrete IL-1β to increase JAG1 expression in astrocytes and thus promote breast cancer stem cell renewal though the Notch signaling pathway [53]. The ECM also participates in the cell communication between primary tumors and metastases [54]. Cancer cells can secrete certain cytokines to stimulate metastatic ECM deposition. Deposition of the ECM in the lung can increase adhesion ability of VLA-4^+^/VEGFR1^+^ hematopoietic progenitor cells (HPCs) in the lungs to stimulate the development of a pre-metastasis into a colonization site for endothelial progenitor cells (EPCs) and disseminated tumor cells (DTCs) [50]. Therefore, how to effectively alter cell communication between primary tumors and metastases would be a new research direction for the prevention and treatment of breast cancer.

### Cell communications between the breast cancer microenvironment and the internal environment

In addition to cell communications between primary tumors and metastases, cell communication can also occur between cells in the microenvironment and cells within other organizations. In the internal environment, proteins, enzymes, hormones, nutrients and metabolites likely act as signaling molecules in cell communication. During lactation, the nerves distributed in mammary glands must transfer signals to the central nervous system, but this process is mainly regulated by hormones secreted from the ovary and pituitary rather than efferent nerves. Therefore, it is meaningful to recognize cell communication mediated by hormones in breast cancer. In addition, metabolites produced by cells within other organs can be treated as generalized signaling molecules that can spread in the local microenvironment and participate in cell communication.

#### Hormones

During the process of mammary gland development and lactation, estrogen, progesterone secreted by the ovaries and prolactin secreted by the pituitary gland play important roles in humoral regulation. The occurrence of breast cancer degradation after ovariectomy suggests that circulating levels of humoral factors regulate the development of breast cancer [27]. The increase in estrogen levels can promote the occurrence and development of breast cancer. Estrogen interacts with estrogen receptor (ER)-positive breast cancer cells and depends on the FGF-FGFR3-TBX3 axis to communicate with breast cancer stem cells to promote their proliferation and dissemination. There are two ways for changes of ER receptor on breast cancer cells. In studies of resistance to endocrine therapies, it has been found that drug-resistant cancer cells have mutations in their ER that enable them to be activated in the absence of ligands [55]. In recurrent breast cancer, a percentage of breast cancer cells can be transformed from estrogen receptor-positive to estrogen receptor-negative cells [56]. In addition to ligand-receptor cell communication, estrogen receptor-negative breast cancer cells also remain under the influence of estrogen. This kind of communication may occur through ER^+^ breast cancer cells stimulated by estrogen and subsequently transmit signaling molecules to influence ER^-^ breast cancer cells. Thus, this is a paracrine-dependent communication mechanism in which estrogen indirectly acts on ER^-^ breast cancer cells [57, 58]. These dynamic changes affect responsiveness of breast cancer patients to endocrine therapy. Researchers have discovered interactions between progesterone gene (PR)-positive and negative breast cancer stem cells through NF-KB (RANK) and its ligand RANKL. The genetic activation of RANK can promote mammary tumorigenesis and proliferation. PRL is the receptor for mammotrophic hormone, which is also expressed in many breast cancers. PRL signaling acts upstream of PR signaling, and thus it can regulate the development of breast cancer by PR. On the other hand, PRL signaling targets the Bcl-6 protein and increases the expression of the transcription inhibitor ZEB1 to suppress the expression of E-cadherin and promote the EMT of breast cancer cells [58]. Because breast cancer occurs more frequently in women, most researchers have focused on the relationship between sex hormone and breast cancer. However, recent findings have shown that other hormones can also influence breast cancer progression, such as thyroxine [59] and adrenaline [60].

#### Metabolites

During the process of tumor progression, tumor cells can acquire many kinds of nutrients via the blood vessels in the tumor microenvironment. However, a variety of metabolites can also be taken up by tumor cells through circulation of the blood and be signaling molecules between cells to regulate several biological behaviors. The tricarboxylic acid (TCA) cycle is one of the important metabolic mechanisms within cells of the body. As an important intermediate in the TCA cycle, alpha-ketoglutarate (α-KG) is not only involved in metabolism, but also serves as a signaling molecule to affect cell proliferation and migration. Increases in α-KG have been found to up-regulate succinate dehydrogenase (SDH) and fumarate hydratase (FH), reduce the onco-metabolites succinate and fumarate, and further stabilize HIF prolyl hydroxylase 2 (PHD2) and decrease HIF-1α, ultimately suppressing breast cancer metastasis [61]. During the decomposition process, fat can be converted into glycerol and fatty acids, including the ω-6 polyunsaturated fatty acids. As a member of these fatty acids, arachidonic acid (ARA) is an important intermediate metabolite in the body. This product can stimulate the ERK1/2 and PI3K/AKT signaling pathways within endothelial cells to promote angiogenesis and metastasis in breast cancer [62]. The Warburg effect is a characteristic of tumor energy metabolism, in which in the presence of normal oxygen content, glucose metabolism is gradually transformed to glycolysis to meet the tumor demand more rapidly for energy. A previous study showed that as one of the metabolic products of glycolysis, lactic acid plays a significant role in the process of cell communications within the breast cancer microenvironment. Peiwen Chen et al. found that lactate can activate G protein-coupled receptor 132 (Gpr132) on macrophages to stimulate macrophage differentiation into the M2 phenotype and promote the adhesion, metastasis and invasion of breast cancer cells [63]. Here, we describe several kinds of cell communication mediated by metabolites in Table 1.

**Table 1:** Indirect cell communication mediated by metabolites

Signal source	Communication molecules	Signal-receiving cells	Functions	References
Circulation	alpha-Ketoglutarate	breast cancer cells	Stabilize HIF prolyl hydroxylase 2 (PHD2) and decrease HIF-1α, ultimately suppressing breast cancer metastasis	[61]
Circulation	Arachidonic acid	Breast cancer cells	Stimulate the ERK1/2 and PI3K/AKT signaling pathways within endothelial cells to promote angiogenesis and metastasis in breast cancer	[62]
Circulation	Lactate	Breast cancer cells	Activate G protein-coupled receptor 132 (Gpr132) on macrophages to stimulate macrophage differentiation into the M2 phenotype and promote the adhesion, metastasis and invasion of breast cancer cells	[63]
Fibroblast	Kynurenine	Breast cancer cells	FORM complex with Aryl hydrocarbon receptor (AhR) to degrade E-cadherin and increase breast cancer invasiveness	[108]
Circulation	Hydroxycholesterol	Breast cancer cells	Contacts estrogen receptor and liver X receptor to promote the growth and metastasis of breast cancer cells	[109]
Circulation	5α-pregnane-3,20-dione	Breast cancer cells	Increases cell proliferation and detachment	[110]
	Lysophosphatidic acid	Breast cancer cells	Contacts lysophosphatidic acid receptor to contribute to the progression of breast cancer	[111]
Circulation	3,3′-diindolylmethane (DIM)	Breast cancer cells	Aborgates tetrachlorodibenzo(p)dioxin (TCDD)-induced inflammation and tumorigenesis	[112]

### Cell communications between the breast cancer microenvironment and the external environment

**Fig. 1:**
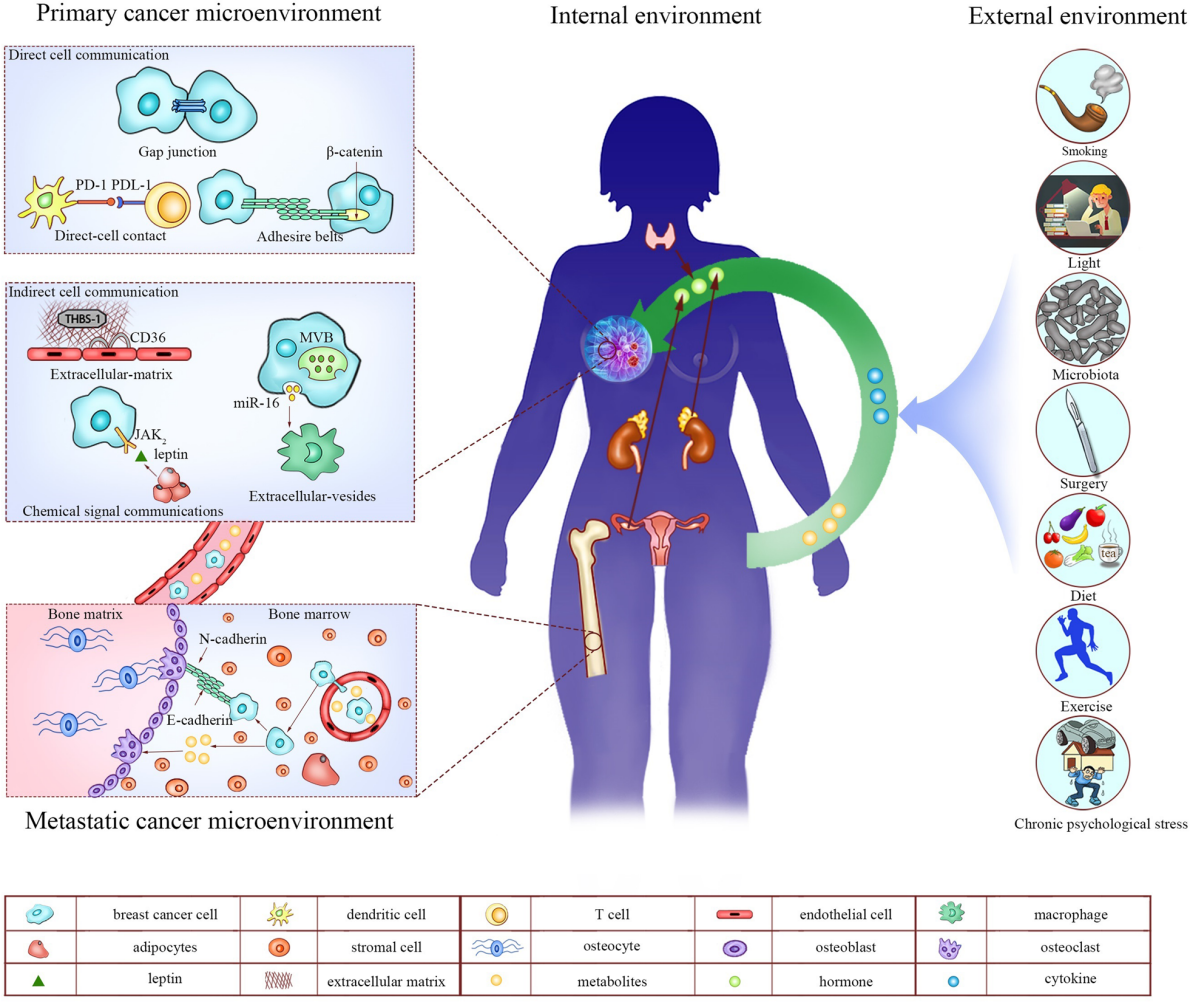
Cell communication exists in primary breast cancer microenvironment, metastatic breast cancer microenvironment, internal environment and external environment and mediates them to communicate with each other

The living environment of the individual is the external environment. Although the external environment cannot directly communicate with the tumor microenvironment, by altering the cells and signaling molecules in the internal environment, external environmental factors can also indirectly affect cell communication in the breast cancer microenvironment. Part of the influence of external environment is more acceptable, and some effects of external factors are dynamic. Therefore, analysis of the external environment involved in cell communications can provide more acceptable strategies for breast cancer treatment [64] (Fig. 1).

#### Smoking

Nicotine is an important carcinogen contained in tobacco products. Tobacco smoking causes most of the nicotine to enter the blood circulation through respiration to the lungs. A study showed that as an important component of the breast cancer microenvironment, fibroblasts are influenced by nicotine. Nicotine can interact with nicotinic acetylcholine receptors (nAChRs) on fibroblasts to promote the expression of CTGF and TGF-β and further promote the EMT and metastasis of breast cancer cells [65]. In vivo studies have shown that smoking can stimulate highly proliferating and undifferentiated mammary epithelium of young nulliparous females to acquire malignancy, but cannot affect the mammary epithelium that has become either differentiated (i.e., after pregnancy and lactation) or quiescent (i.e., after menopause) [66]. These contradictory results indicate that the influence of smoking on the body may be related to the local microenvironmental state of breast epithelial cells and that the outcome is alterable.

#### Light

A study showed that circadian rhythm disorder is a risk factor for breast cancer. In the human body, the circadian rhythm is produced by the endogenous circadian clock. Research has suggested that the biological clock is mainly controlled by the light cycle. One study showed that compared with normal daytime workers, night time workers have an increased incidence of breast cancer by 10%–60% [67]. In 1987, the first suggestion was proposed that light at night might explain a portion of the breast cancer pandemic. That finding was based on the idea that exposure to light at night would result in melatonin suppression, which in turn would increase breast cancer risk [68]. T-helper cells play an important role in protection against malignancy. Melatonin is a natural antioxidant with immuno-enhancing properties, and it has been shown to enhance the T-helper cell response by releasing IL-2, IL-10 and IFN-γ. Melatonin is effective in suppressing neoplastic growth in breast cancer [69]. Light of the blue region of the spectrum shows the strongest suppression of nocturnal melatonin, which suggests that people who often work at night and under light have a weakened immune ability and higher risk of breast cancer. Optical and lighting devices that filter the blue light spectrum have been proposed to avoid the light-induced suppression of nocturnal melatonin and have been applied to prevent breast cancer [70]. The use of melatonin can also counter the light-induced increased risk of breast cancer [71].

#### Microbiota

Breast tissue is not sterile but contains a diverse population of bacteria [72]. Notably, breast cancer has a richer and more diverse microbiome than lung cancer, melanoma, pancreas cancer [73]. While researchers have reported differential abundances of certain organisms between healthy and diseased states, in reality, a single organism is likely not responsible for driving disease progression or protection but an interplay of polymicrobial interactions [74]. Compared with normal breast tissue, higher relative abundances of Enterobacteriaceae, Bacillus and Staphylococcus epidermidis and Staphylococcus Escherichia coli (a member of the Enterobacteriaceae family) in breast cancer, were found to induce DNA double-stranded breaks in HeLa cells [72]. An epidemiological study has shown that women who drink fermented milk products have a reduced risk of breast cancer development, irrespective of multivariable risk factors. This protection might be attributed to the health-promoting properties of the various lactic acid bacteria (LAB) present in fermented products. Lactococcus and Streptococcus, two such bacteria that are more abundant in healthy women than in breast cancer patients, exhibit anti-carcinogenic properties and may play a role in prevention. Natural killer (NK) cells are vital in controlling tumor growth, with epidemiological studies showing that low NK cell activity (from peripheral blood mononuclear cells [PBMC]) is associated with an increased incidence of breast cancer. Lactococcus lactis has been shown to activate murine splenic NK cells, enhancing cellular immunity. Lactococcus sp. present in mammary glands may modulate cellular immunity by maintaining the cytotoxic activity of resident NK cells, thus helping to prevent cancer development. Streptococcus thermophilus, in contrast, protects better than any other LAB tested against DNA damage caused by reactive oxygen species by producing antioxidant metabolites that neutralize peroxide and superoxide radicals [72]. In addition to the microbes of mammary gland tissue, the microbes of other tissues also affect the development of breast cancer. When estrogen is metabolized through the liver and excreted from the urine or intestinal tract, the conjugated estrogens excreted in the bile can be deconjugated by the microbes in the intestinal tract with β-glucuronidase activity, and then reabsorbed by the body through the enterohepatic circulation, acting on target organs such as the breast, affecting the progress of the disease [75]. Soy-based isoflavone can be metabolized by gut microbiota into equol, and the increase of equol is associated with a reduced risk of breast cancer in Asian women [76]. There remain controversies about the relation among soy intake, the equol-producer phenotype, the concentration of equol and the risk of breast cancer. These findings suggest the potential in this area and the value of exogenous food or microorganisms to regulate host microbiome status to provide more efficient and acceptable therapeutic strategies [77].

#### Surgery

Surgical treatment is an important strategy to cure breast cancer. For patients, surgical treatment not only includes removal of the primary tumor, but it can also alter cell communication between local tissue and distant metastases. Primary tumors in breast cancer can block the angiogenesis of micro-metastatic foci and produce metabolic byproducts to inhibit proliferation. Removal of the primary tumor can eliminate these inhibitions and avoid competition for essential host-derived nutrients that are required for tumor proliferation between the primary tumor and its metastases [78]. High levels of mitogenic and angiogenic factors and low levels of antiangiogenic factors are observed in fluid from mastectomy wounds, suggesting that local inflammation and wound healing induced by surgery can promote the growth of tumor cells. Additionally, surgical treatment can increase circulating level of immuno-suppressive factors induced by surgical stress in patients [79]. Decreases in the numbers of circulating cytotoxic T lymphocytes, NK cells, T-helper cells, and dendritic cells have been observed after surgery. Moreover, the production of cytokines, that favor cell-mediated immunity (e.g., IL-2, IL-12 and IFN-γ), is also decreased. Therefore, removal of the primary breast cancer is not always beneficial.

#### Chronic psychological stress

In modern society, the quickening pace of life has introduced high-stress work, disordered daily schedules, and social environmental pressures, resulting in a state of long-term psychological stress. Epidemiological studies have shown that compared with women living in the countryside, women living in the faster pace of urban life have a higher incidence of breast cancer. Hongyu Chen found that mice living under a chronic stress state more easily developed lung metastasis of breast cancer. However, using propranolol to block β-adrenergic signaling can observably suppress stress-induced lung metastasis. The underlying β-adrenergic mechanism induces the generation of monocytes and macrophages that metastasize to the lung to promote pulmonary metastasis of breast cancer cells [60]. In summary, the social environment can play a role in the regulation of nerves and hormones and, depending on the internal environment, further affect development of the local breast cancer microenvironment.

#### Diet

Eating habits are often associated with the development of cancer. Lutein is an important component of plant pigments in corn, vegetables, and fruits. As a kind of carotenoid, lutein is present in the leaves of the plant chloroplast, and the body can acquire it through the daily intake of fruits and vegetables. In recent years, lutein has been shown to increase the content of active oxygen to inhibit the proliferation of triple-negative breast cancer cells [80]. In postmenopausal women, adopting a low-fat diet, including the increased intake of vegetables, fruits and grains, may reduce the mortality of breast cancer [81]. Interestingly, in hormone receptor-positive breast cancer patients, periodic fasting or a fasting-mimicking diet can reduce the circulating levels of insulin-like growth factor 1 (IGF1), insulin and leptin by inhibiting the AKT-mTOR signaling pathway. That can enhance the activity of endocrine therapy including tamoxifen and fulvestrant [82]. In addition to food, the composition of daily drinks also affects the occurrence and development of breast cancer. Shu-min LIU et al. found that green tea polyphenols (GTPs) can induce cell cycle arrest and mitochondria-mediated apoptosis (induction of DNA fragmentation, improved generation of reactive oxygen species, induction of chromatin condensation) to suppress the development of breast cancer [83]. Curcumin, genistein and agents can affect histone acetylation and methylation. This has attracted the attention of more researchers because epigenetic agents have the potential to overcome drug resistance [84]. These findings suggest that a reasonable dietary plan according to body condition can be used as an adjuvant to prevent and treat breast cancer.

#### Exercise

It is widely believed that regular exercise habits are beneficial to health. Researchers found that resistance training can decrease inflammatory factors and is associated with prolonged survival for breast cancer patients [85]. A paper has suggested that appropriate exercises can reduce the incidence of tumors and slow tumor growth [86]. In Christine Dethlefsen’s study [87], sera were extracted from normal women and breast cancer patients with regular exercise training and were then used to treat breast cancer cells respectively. They found that both could significantly reduce the viability of breast cancer cells, and the same results were obtained in a mouse model. Further analysis showed that exercise could improve catecholamine (epinephrine and norepinephrine) levels and inhibit the proliferation and survival ability of breast cancer cells by affecting the Hippo signaling pathway and down-regulation of target genes (such as the expression of ANKRD1 and CTGF).

## Dynamic cell communication in the breast cancer microenvironment

The dynamic changes of cell communication in the breast cancer microenvironment are closely related to the changes in communicating cells and molecules. These changes can be attributed to the changes in communication induced by molecules in the external environment, as well as the internal differences in the composition and heterogeneous functions of communication cells and molecules. Moreover, the stage of occurrence and progression of breast cancer greatly enrich the connotation of this dynamic change. Therefore, the concept of dynamic cell communication should be deeply rooted and implemented in the clinical diagnosis and treatment of breast cancer, to improve the therapeutic effect.

### Dynamic changes in the communicating cells in the breast cancer microenvironment

With the development of research examining the breast cancer microenvironment, the medical community is increasingly focusing on immuno-therapeutic strategies and elucidation of the dynamic changes in immune cells. Moreover, immune cells play important roles in communication between the external environment and the microenvironment. Therefore, tracking the dynamic changes in immune cells is of great significance. In general, the dynamic changes in the immune cells in the breast cancer microenvironment can be classified based on three conditions. A. The same immune cells with different functions during different stages of tumor development. During different inflammatory conditions in the breast cancer microenvironment, the functions of immune cells are significantly different. Acute stimulation of T lymphocytes leads them to differentiate into Th1 cells and secrete INFγ, TNFα and IL-2 to synergistically inhibit cancer development with CD8^+^ T cells. In this way, cancer cells are phagocytized or destroyed by innate immune cells. However, chronic inflammatory stimulation can promote T cells to differentiate into Th2 cells, which secrete IL-4, IL-5, IL-6, IL-10 and IL-13. This will induce T-cell anergy and the loss of T-cell-mediated cytotoxicity, thus promoting the progression of cancer [88]. B. The same immune cells with dual functions. It is known that regulatory T cells can suppress immune responses, but the survival rate of breast cancer patients will improve if a high level of Tregs is present in breast cancer tissue. These observations suggest that Tregs play a dual role in cancer. Tregs promote macrophages and neutrophils to produce IL-1, IL-6, IL-23, TNF-α and activate Th17 lymphocytes to produce IL-17, IL-21 and IL-22. These cytokines act on cancer cells to drive their proliferation and survival. On the other hand, Tregs can also control mechanisms that facilitate the antigen presentation of dendritic cells (DCs) and Th1 cells to further activate NK cells and cytotoxic T lymphocytes (CTLs) [89]. The ultimate outcome of Treg immune modulation in cancer development depends on the relative strength and efficiency of their pro- and anti-tumor immunity systems [90]. C. The proportions of immune cell subtypes with different functions, for example, tumor-associated macrophages, can be divided into multiple cell subtypes based on molecular markers and functions, including M1, M2a, M2b and M2c. In different stages of breast cancer development, the ratio of different subtypes of macrophages infiltrating into the microenvironment are different. In the early stage of breast cancer, most macrophages are M1, which are mainly involved in pro-tumor immunity. In advanced breast cancer, most macrophages are M2, which can promote tumor growth and metastasis [88]. The reason for this dynamic change is partly due to the cellular communication with breast cancer cells. Tumor-associated macrophages (TAMs) co-cultured with estrogen receptor (ER)-positive/luminal breast cancer cells can acquire the M1 phenotype to suppress cancer, and those co-cultured with triple-negative/basal breast cancer cells can acquire the M2 phenotype to promote cancer [91]. Studies have also demonstrated the same situation in tumor-associated neutrophils (N1 and N2 types) [92] and T cells (Th1 and Th2 types) [88], fully reflecting that the changing proportions of different immune cell subtypes can regulate the occurrence and development of breast cancer. Now, strategies for breast cancer treatment are based mainly on the molecular characteristics of the primary tumor, but usually the efficacy is not ideal. Researchers have found that at the genetic level, there is significant heterogeneity between primary and metastatic breast cancers, which may affect the efficacy of the drug [93]. However, when other researchers compared the molecular markers between the primary and metastatic breast tumors at the cellular level, no significant differences were found [94]. In addition, we should notice the important role of the complement signal. After patients received chemotherapy for breast cancer, ICOSL^+^ B cells, which elicit anti-tumor T cell immunity and improve therapy efficacy, emerged [95]. Therefore, the cellular changes during the progression of breast cancer deserve further study.

### Dynamic changes in communication molecules in the breast cancer microenvironment

The dynamic changes in the breast cancer microenvironment are closely associated with changes in various communication and signaling molecules. These changes include the structure, content, and location of signaling molecules and the complexes formed with other molecules. Any slight alterations could change or even reverse the existing pattern of cell communication. These molecules such as the adhesion molecules PD-L1 and PD-1, which mediate direct communication between tumor cells and T cells, VEGF and VEGFR, which mediate indirect cell communication in a paracrine and autocrine manner, and estrogen and estrogen receptors, which mediate indirect cell communication similar to hormone and lactic acid metabolite mediation of indirect communication in the local acidic microenvironment, are often considered to be targets in breast cancer treatment. Among these molecules, as the most important molecules in immune therapy, immuno-suppressive molecules have engaged the attention of researchers. In investigations ofimmune escape, researchers have discovered that these immuno-suppressive molecules can change during breast cancer development or in different subtypes of breast cancer.

Immuno-suppressive molecules refer to molecules that can inhibit the immune response and induce immune evasion. The main immuno-suppressive factors associated with breast cancer include PD-L1, GM-CSF, CTLA4, SDF1, CXCL5, IDO, IL-4, IL-8, IL-10, IL-13, MIF, NOS2, PD1, COX2, VEGF and TGF-β. Most of these immuno-suppressive factors mainly suppress the immune response and promote cancer development in a cell-cell communication manner. In addition, some molecules play different roles during breast cancer development. There are two major sources of TGF-β: mononuclear cells (precursors for macrophages) and low-density polymorphonuclear cells (immature neutrophils) [96]. Takeshi Imamura divided cancer development into the early stage and late stage and found that TGF-β signaling has two distinct and opposite roles in cancer progression and metastasis. During the early stage of carcinogenesis, TGF-β strongly suppresses cell growth and acts as a tumor suppressor. In contrast, in the late stage of cancer progression, TGF-β induces the EMT and invasion of cancer cells and accelerates metastasis [97]. IL-10, a well-established suppressor of immunity, reduces the antigen presentation capacity of macrophages and inhibits the production of several cytokines that have important roles in tumor immuno-surveillance. Therefore, elevated levels of IL-10 may facilitate tumor immune escape [98]. However, IL-10 has been shown to have anti-tumor properties. The proposed anti-cancer activity mechanisms of IL-10 include the activation of NK cells, enhancement of the surface expression of the MHC antigen to maintain the susceptibility of cancer cells to NK cells, the synergistic activation of CTL for maintenance of the CD8^+^ and CD4^+^ mediated anti-tumor response, the modulation of angiogenesis and invasiveness through the inhibition of metalloproteinase, and finally, the enhancement of tumor infiltration by neutrophils and macrophages. In addition to changes in the functions of molecules, the content of molecules also affects their final roles in breast cancer cells. That is particularly important in clinical chemotherapy for breast cancer. It has been found that the maximum-tolerated dose of standard breast cancer chemotherapy drugs (doxorubicin, paclitaxel, or 4-hydroxy-cyclophosphamide) can promote the proliferation of cancer cells by acting on CAFs, and may even reverse the anticancer effects of chemotherapy drugs. Some researchers have suggested that low-dose metronomic therapy could reduce this effect and increase the effect of the drug [99]. In addition, the efficacy of some treatments is influenced by dynamic cell communications (Table 2).

**Table 2:** Therapeutic effects influenced by dynamic cell communications

Treatment	Changes in effects	References
Surgical removal of the primary cancer	Eliminate inhibitions from primary tumors to micro-metastatic foci	[78]
Target of ER	Mutations in ER or transformations from ER^+^ to ER^−^	[55]
Doxorubicin	Maximum-tolerated dose of doxorubicin promotes the proliferation of cancer cells	[99]
Microwave ablation combined with OK-432	Change the ratio of Th1 to Th2 cells to reduce reoccurrence of cancer	[113]
Target of metastatic cancer	Heterogeneity between the primary and metastatic breast cancer	[93]
Neoadjuvant versus adjuvant chemotherapy	Patients receiving neoadjuvant chemotherapy have a higher local recurrence rate	[114]

## Discussion

Cell communication and signal transduction are processes that coordinate various communication molecules in vitro, in vivo and in the microenvironment, and finally produce integrated reactions in target cells. Tumors are characterized by the abnormal proliferation and differentiation of target cells and represent an adaptive response to various upstream communication related cell and molecular variations. When we fully realize and enhance the significance of cell communication research, there are still some problems in the research field of dynamic changes of breast cancer cell communication.

### More accurate individual treatment based on dynamic cell communications in breast cancer

Tumor size, metastasis to lymph nodes, distant organ metastasis or ER, PR and ERBB2 are usually used as indicators for the clinical classification of breast cancer. These methods cannot satisfy the needs of treatment. Analogous to surgical treatment, the current criteria for the stages of cancer and treatment efficacy cannot explain how the primary cancer promotes the development of metastatic cancer. In the future, these criteria will restrict advancements in the clinical diagnosis and treatment of breast cancer. Taking breast cancer metastasis as an example, current breast cancer staging is heavily dependent on the evaluation of pathology specimens, in which the index of lymph node metastasis or organ metastasis is clearly very uncertain. However, as described earlier, using specific signaling pathways and gene expression profiles may further classify triple negative breast cancer, and Liu Y et al. recently divided the formation of the pre-metastatic niche into 4 stages: priming, licensing, initiation and progression, and enhanced the classification of phenotypes and stages of breast cancer [100]. Moreover, recent findings support that the functional classification of breast cancer may become an important addition to risk prediction and prognosis. As determined by their ability to promote the outgrowth of micro-metastatic tumor populations at distant sites, preclinical animal models suggest that it might be possible to classify breast cancers on a functional basis [101]. Up to 5% of patients need to receive a variety of high cytotoxic treatments because they cannot be diagnosed about the primary origin of carcinoma. Fortunately, it has been found that different cancers including breast cancer, colorectal cancer and mesothelioma can be distinguished by detecting specific extracellular vesicles and particles (EVP) in cancer tissue or plasma [102]. Moreover, research on cell communication can promote the development of proteogenomics to help identify tumor biology that is otherwise undiscernible using genomic profiles alone, such as non-mutational “epigenetic” causes of inactivation and posttranslational modifications [103]. Thus, the use of dynamic cell communication as a new molecular marker to divide breast cancer into different stages is feasible and has great significance.

### External environmental factor-oriented treatment strategies for breast cancer

We are influenced by the external environment in which we always live. Moreover, most of these influences are easy to acquire. Similar to changing the local immune microenvironment, intervening in external environmental factors combined with conventional therapies can undoubtedly produce better effects of cancer therapy. Many external environmental factors can influence the overall physiological state of the body (such as smoking, light, the microbiota, exercise, diet, and alcohol [104]). These changes of body can further affect the local microenvironment, especially by changing breast cancer cell communications to affect breast cancer development. Fortunately, some external environmental interventions are easily accepted. The latest research suggests that the use of external environment factors is of great significance as a supplementary means of cancer treatment [105]. It has been reported that L-thyroxine functions similar to conventional clinical drugs for thyroid disorders in patients with breast cancer. The use of L-thyroxine to treat breast cancer patients undergoing chemotherapy can promote body fatigue and further increase the risk of cancer development. However, after an exercise training intervention, the fatigue condition associated with the use of L-thyroxine will be significantly improved. Therefore, researchers have recommended that breast cancer patients who receive chemotherapy with L-thyroxine participate in exercise training to achieve a better therapeutic effect. Therefore, guided by interventions in external environment factors, a more efficient, economic and acceptable breast cancer prevention and treatment strategy is possible.

### Recessive cell communication merits more attention in basic research

The ultimate goal of cell communication is to induce downstream target cells to produce a variety of biological effects in response to changes in external environmental factors. In these biological effects, we often focus on communicating cells with a dominant effect on biology, such as rapidly proliferating cells, dysfunctional cells, and cell communities that exhibit explosive growth during metastasis, but ignore the communication between cells that do not produce obvious pathological changes, termed "recessive cell communication". Two of these cell types and their associated microenvironments are worth mentioning. One type is the temporary dormant cell in a relatively suitable metastatic microenvironment (i.e., the highly metastatic organs in breast cancer), and another type is the long-term dormant cell in a relatively inappropriate metastatic microenvironment (i.e., organs with low metastatic potential or organs that are not metastasized in breast cancer) [106].Tumor dormancy is the result of equal rates of cell proliferation and cell death or a slow rate of proliferation due to immune surveillance or poor blood perfusion. Cell communication also exists between dormant cells and the microenvironment as well as the internal environment, which do not exhibit dominant biological effects. For example, Wen et al. found that as organs that are easily metastasized, the lung and liver have more myeloid-derived suppressor cells and an accumulation of exosomes, while the bone shows no changes in immune cell composition and no significant accumulation of exosomes [107]. These findings suggest that cell communication also shows metastatic organotropism to induce different effects. Concomitantly, the present stage of breast cancer treatment strategies mostly consists of targeting the dominant effect of cell communication; however, the side effects and long-term effects of this clinical treatment are not optimistic. In comparison, "recessive cell communications" are more similar to the natural condition of the body and greatly maintains the steady state of the internal environment. Therefore, this kind of naturally occurring cell communication not only has important value in research, but also provides new ideas and directions for the clinical treatment of breast cancer.

## Conclusion

- 1. Cell communication in the breast cancer microenvironment includes direct and indirect ways and all the communication patterns are dynamic. Understanding the dynamic changes in the microenvironment of breast cancer is very important for the individualized diagnosis and treatment of breast cancer.
- 2. External environmental factors and internal environmental signaling molecules can communicate with the primary local microenvironment of breast cancer. Guided by interventions involving external environmental factors, a more efficient, economic and acceptable breast cancer prevention and treatment strategy is possible.

## Data Availability

The datasets support the conclusions of this study. Any requests for data can be sent to the author.

## Abbreviations

ECM: Extracellular matrix
EMT: Epithelial-mesenchymal transition
α-KG: alpha-Ketoglutarate
Gpr132: G protein-coupled receptor 132
TAMs: Tumor-associated macrophages
